# Kappa but not delta or mu opioid receptors form homodimers at low membrane densities

**DOI:** 10.1007/s00018-021-03963-y

**Published:** 2021-10-17

**Authors:** Kristina Cechova, Chenyang Lan, Matus Macik, Nicolas P. F. Barthes, Manfred Jung, Maximilian H. Ulbrich

**Affiliations:** 1 Department of Biomathematics, Institute of Physiology, Czech Academy of Sciences, Prague, Czech Republic; 2 Department of Biochemistry, Faculty of Science, Charles University, Prague, Czech Republic; 3 Faculty of Biology, University of Freiburg, Freiburg, Germany; 4 Prague, Czech Republic; 5 Institute of Pharmaceutical Sciences, University of Freiburg, Freiburg, Germany; 6 CIBSS-Centre for Integrative Biological Signalling Studies, University of Freiburg, Freiburg, Germany; 7 Institute of Internal Medicine IV, Medical Center of the University of Freiburg, Freiburg, Germany; 8 BIOSS Centre for Biological Signalling Studies, University of Freiburg, Freiburg, Germany

**Keywords:** Single-molecule imaging, Dimerization affinity, Opioid receptors, G protein-coupled receptors, Monomer-dimer equilibrium

## Abstract

Opioid receptors (ORs) have been observed as homo- and heterodimers, but it is unclear if the dimers are stable under physiological conditions, and whether monomers or dimers comprise the predominant fraction in a cell. Here, we use three live-cell imaging approaches to assess dimerization of ORs at expression levels that are 10–100 × smaller than in classical biochemical assays. At membrane densities around 25/µm^2^, a split-GFP assay reveals that κOR dimerizes, while µOR and δOR stay monomeric. At receptor densities < 5/µm^2^, single-molecule imaging showed no κOR dimers, supporting the concept that dimer formation depends on receptor membrane density. To directly observe the transition from monomers to dimers, we used a single-molecule assay to assess membrane protein interactions at densities up to 100 × higher than conventional single-molecule imaging. We observe that κOR is monomeric at densities < 10/µm^2^ and forms dimers at densities that are considered physiological. In contrast, µOR and δOR stay monomeric even at the highest densities covered by our approach. The observation of long-lasting co-localization of red and green κOR spots suggests that it is a specific effect based on OR dimerization and not an artefact of coincidental encounters.

**Supplementary Information:**

The online version contains supplementary material available at 10.1007/s00018-021-03963-y.

## Introduction

ORs are G protein-coupled receptors (GPCRs) from class A with three genes coding for the δOR, κOR, and µOR. The µOR is the most prominent clinical target for pain medication. However, side effects of opiates like respiratory depression and the potential for opioid addiction have increased the efforts to develop drugs with novel pharmacological profiles. Also, it has become clear in recent years that differential control of downstream mechanisms (G protein activation vs. β-arrestin recruitment) or restriction of drugs to the peripheral nervous system allows to further reduce unwanted side effects. Therefore, improving our understanding of OR activation and signaling will support the development of novel treatments.

Based on functional characterization of ORs, more subtypes than δ, κ, and µ were proposed, which can be explained by the existence of splicing variants, posttranslational modifications and/or direct interactions between receptors. Dimerization of ORs has been covered in multiple studies, yet the conclusions were contradictory, as for many other GPCRs, likely due to the use of differing methodological approaches and experimental conditions [1]. For all three ORs, dimerization has been observed [2–6]. So far, the major techniques to assess dimerization of ORs were co-immunoprecipitation followed by Western blotting, and bioluminescence resonance energy transfer (BRET). Both are bulk techniques, where the signal is obtained from a large population of cells. Not only is the major part of the signal caused by a small, highly expressing fraction of the cells, but in addition, these few cells express the receptors at the highest density. Therefore, the signal mainly reflects the receptor's behavior at a membrane density that is far above the physiological range, and cannot capture the state of the receptors at low membrane densities, as they prevail in many cells in vivo.

To assess specific interactions of membrane proteins that are likely to exist in vivo as well, single-molecule imaging in living cells is the perfect tool. At densities around 0.1–1/µm^2^, single fluorescently labeled molecules can be tracked, and dimerization or higher-order cluster formation can be deduced from co-localization of differentially labeled receptors or from the analysis of intensity histograms from many individual receptor complexes [7, 8]. For several class A GPCRs, single-molecule imaging suggested the existence of dimers [7, 9, 10]. µOR seems to be monomeric when unliganded and switches to a dimeric state when the ligand DAMGO is bound, but interestingly stays monomeric upon binding of morphine [11, 12]. κOR has been observed to be primarily monomeric [13]. However, for these studies, experiments were performed at low densities < 1/µm^2^. A recent study using single-molecule FRET also observed unliganded µOR to be monomeric at a density of up to 4/µm^2^, and also at densities around 100/µm^2^ using pulsed-interleaved excitation fluorescence cross-correlation spectroscopy (PIE-FCCS) [14]. No study so far investigated dimerization of the δOR at low densities.

The observation of unliganded OR monomers at low densities and dimers in bulk experiments supports the notion that dimerization is primarily driven by association and dissociation of receptors at the plasma membrane, which at higher densities, shift the equilibrium to a higher dimer fraction. In this work, we, therefore, set out to investigate OR dimerization at densities that were not covered previously, with the goal to determine the dissociation constant, i.e., the density at which the switch from monomers to dimers occurs. To this end, we measured OR dimerization using three microscopy approaches that work at different densities. Our starting point was a split-GFP fluorescence complementation assay in cells with expression levels in the range of 10–100/µm^2^. This assay gave us a first indication that the κOR has a tendency to dimerize, while the δOR and µOR resembled the monomeric control, which was the PDGFRα transmembrane domain (PDGFRTM) [15–17]. With conventional dual-color single-molecule imaging at densities below 5/µm^2^, we did not observe a significantly higher co-localization of green and red labeled proteins for κOR than for the other ORs or a monomeric control. To further investigate the dimerization at higher densities, we turned to a recently developed technique called PhotoGate that allows tracking of single molecules in an originally crowded environment by controlling the density of fluorescent molecules in a region of interest [18]. We quantified the monomer/dimer ratio at densities of up to 150/µm^2^, a level that is thought to be in the physiological range for many GPCRs. δOR and µOR remained predominantly monomeric at all densities tested, whereas κOR formed dimers at higher levels, with a dissociation constant of *k*_d_ = 32 ± 15/µm^2^.

## Results

### Split GFP complementation suggests a higher tendency of κOR to dimerize

To assess dimerization of ORs, we used fluorescence complementation of a split GFP. Here, two fragments of GFP, which on their own are not fluorescent, are fused to the target proteins. Upon encounter, the two fragments covalently bind to each other, which allow the GFP chromophore to form and fluorescence to recover [19]. In our case, we would co-transfect an OR fused to the first fragment with the same OR fused to the second fragment; in case of dimerization of the OR, fluorescence of the reconstituted GFP should appear.

One known disadvantage of the split-GFP assay is that also non-interacting target proteins can lead to a certain degree of GFP complementation due to spontaneous encounters of the two fragments. Another problem is that fluorescence recovery is not proportional to the degree of dimer formation because once the two parts are connected, they cannot separate again. Both effects impede an accurate quantification of the dimerization process, and, therefore, the GFP complementation assay only allows a qualitative assessment of dimerization. Nevertheless, due to its simplicity, this assay has frequently been used [20].

**Fig. 1:**
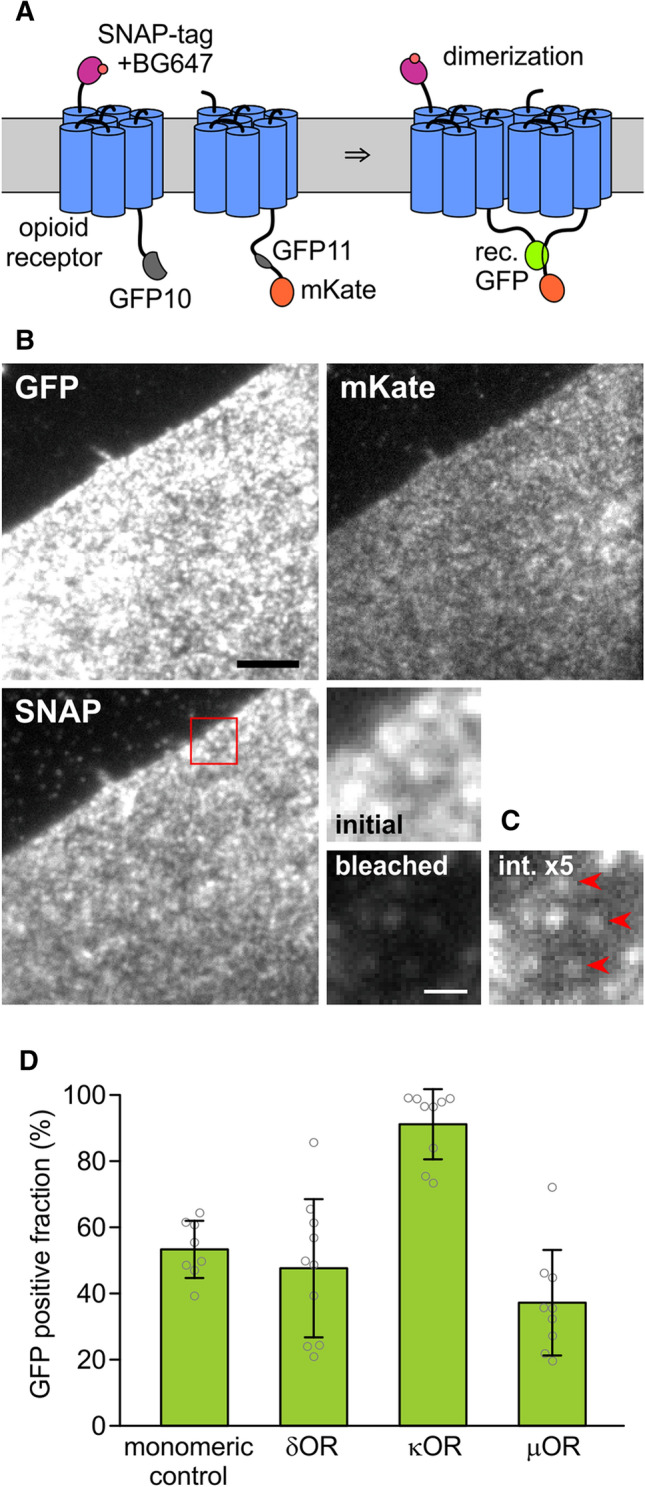
Split GFP assay for opioid receptor dimerization. **A** To determine the densities of the two differentially labeled OR subunits, one is labeled with a SNAP-tag and its far-red fluorescent substrate BG647, and the other with mKate which emits in the orange/red range. They are also labeled with two GFP fragments. Dimerization is detected by fluorescence recovery through complementation of the split GFP. **B** Images of a CHO cell expressing SNAP647-κOR-GFP10 and κOR-GFP11-mKate in the three channels. The red square marks the magnified area. Scale bar 5 µm. **C** Magnified area: molecular densities were determined by measuring the initial fluorescence, bleaching the cell, and then measuring the intensities of remaining single molecules. For the last image, the brightness is increased by a factor of 5. Red arrowheads mark single molecules. Scale bar 1 µm. **D** The GFP-positive fraction for the ORs and the monomeric control was calculated from apparent densities of SNAP647, mKate, and reconstituted GFP

Out of several different previously used split positions, we chose the one between the 10th and 11th beta strand of the GFP barrel, yielding one fragment containing the first ten strands (GFP10), and one fragment containing the last strand (GFP11) [21]. To determine the fraction of split-GFP tags that recover fluorescence upon binding, we need to know the densities of the fragments and the density of recovered GFP. To this end, the two constructs carrying GFP10 and GFP11 were additionally tagged with mKate, which emits in the orange range, and the SNAP-tag, which we labeled with a far-red substrate (Fig. 1A). By comparing the intensities emitted from highly expressing cells with intensities of single molecules of GFP, mKate and the far-red labeled SNAP-tag, we calculated the absolute densities of these species in the membrane.

We generated pairs of δOR, κOR, or µOR, or the PDGFRTM as a monomeric control, fused with an N-terminal SNAP-tag and C-terminal GFP10, or C-terminal GFP11 and mKate, respectively, resulting in SNAP-X-GFP10 and X-GFP11-mKate, where X was δOR, κOR, µOR, or PDGFRTM (Fig. 1A). CHO cells expressing a matching pair were labeled with the far-red fluorescent dye Alexa Fluor 647 conjugated to benzylguanine (BG647), and imaged with 488 nm, 561 nm, and 637 nm excitation in Total Internal Reflection (TIR) configuration (Fig. 1B). From highly expressing cells (densities 7–90/µm^2^, average 20–26/µm^2^) with a similar density of mKate and BG647-labeled SNAP-tag (SNAP647), we recorded a snapshot for each wavelength range to determine the fluorescence intensity per area. For each of the three channels, single-molecule intensities were measured from nearly photobleached or low-expressing cells where individual, diffusing molecules were visible that photobleached in a single step (*n* ≥ 10 for each channel, Fig. 1C and Suppl. Fig. 1).

The molecule densities of SNAP647, mKate, and (reconstituted) GFP tags were calculated from the ratios of intensities per area in the highly expressing cells to the intensities of single molecules from the low-density cells. The GFP-positive fraction was calculated as the ratio of GFP density to the smaller value of mKate and SNAP647 densities (for the rationale behind this approach, see Suppl. Note 1). For the monomeric control, the GFP-positive fraction was 53 ± 9% (s.d., *n* = 8), and the smaller one of the mKate and SNAP647 surface density 23 ± 18/µm^2^, for δOR 48 ± 21% (26 ± 23/µm^2^, *n* = 10), for κOR 91 ± 11% (26 ± 22/µm^2^, *n* = 9), and for the µOR 37 ± 16% (20 ± 14/µm^2^, *n* = 9) (Fig. 1D).

Despite the PDGFRTM is thought to be monomeric, it also had a sizeable GFP-positive fraction due to reasons discussed above. The significantly larger GFP-positive fraction (*p* < 0.00007) for the κOR than for the monomeric control, δOR, and µOR suggests that the κOR has a higher tendency to dimerize than the other two ORs. The GFP-positive fractions for δOR (*p* < 0.63) and µOR (*p* < 0.02) are more similar to monomeric control, which gave us an indication that they might be monomeric as well, which would confirm results obtained for µOR in other studies [11, 12, 14].

### Single-molecule imaging does not show more dimers for κOR than for δOR and µOR

The split-GFP approach indicated an increased dimerization tendency for κOR than for δOR and µOR, but an accurate quantification using this approach is impossible due to the stickiness of the GFP fragments. Also, the efficiency of GFP fluorescence recovery upon complementation is unknown, and, therefore, the split-GFP approach can only deliver a qualitative description. For these reasons, we chose a more direct approach and imaged green and red labeled receptors in the cell membrane on a single-molecule level. When the density of receptor molecules is sufficiently low to yield a large separation distance, then co-localization caused by random encounters is small, and the main cause for co-localizing green and red spots should be the dimerization. However, with increasing spot density, the contribution from random overlap to co-localization increases, and, therefore, we will account for this effect in the calculation of dimer fraction from the degree of co-localization. Since we abstained from the use of the split GFP and also introduced the mutation A206K that virtually eliminates the tendency of GFP to dimerize, the dimer fraction of the ORs should not be affected by interactions of the tags [22].

**Fig. 2:**
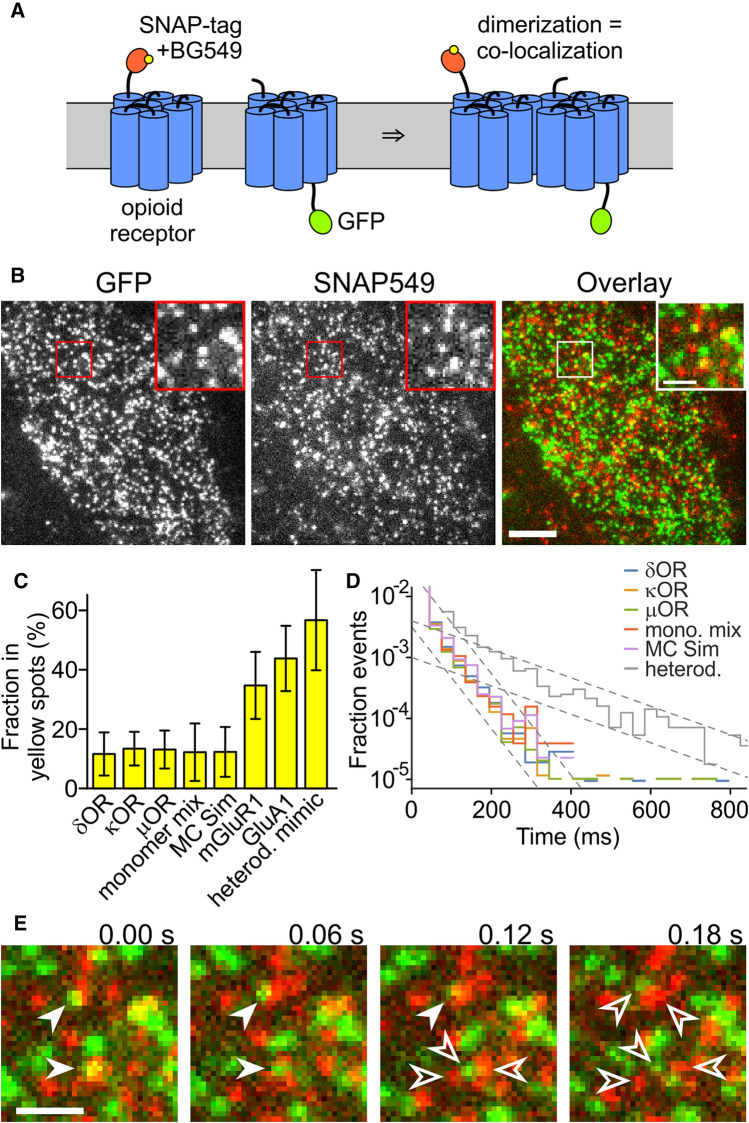
OR co-localization imaged on a single-molecule level. **A** One receptor is labeled with the SNAP-tag and BG549, the other with GFP. When the receptors dimerize, the green and orange-red fluorescence co-localize. **B** Still image from a dual-color movie. Inset shows magnified area. Scale bar 5 µm (inset 1 µm). **C** The fraction in yellow spots is similarly low for all ORs, the monomer mix, and the MC simulation of mixed monomers. For mGluR1, GluA1, and the heterodimer mimic, the fraction of yellow spots is higher. **D** The histogram of co-localization times decays with a time constant of about 30 ms for all proteins and the MC simulation but 120 ms for the SNAP549-PDGFRTM-GFP. **E** Green and red spots that overlaid to yellow (filled arrowheads, here an example from µOR) moved apart within a few frames due to diffusion (empty arrowheads). Scale bars 1 µm

We fused a C-terminal monomeric GFP or an N-terminal SNAP-tag to the target proteins, hereby obtaining X-GFP and SNAP-X, where X was δOR, κOR, µOR, or PDGFRTM (the monomeric control). The SNAP-tag was labeled with a conjugate of the orange–red dye DY-549P1 and benzylguanine (SNAP549) (Fig. 2A). We recorded 488 nm/561 nm excitation dual-color movies of CHO cells expressing one of the X-GFP/SNAP549-X pairs (Fig. 2B and Suppl. Movies 1, 2). We imaged cells with low-surface densities of < 5/µm^2^ of the proteins because with higher expression, individual molecules could not be separated from each other and random co-localization of the green and red emission from GFP and SNAP549 became too high. For all four protein pairs, we observed red and green fluorescent spots, and in a minor fraction of spots, red and green signals overlapped. The mobility of the spots was in the range described for ORs and other GPCRs in previous studies, and only a small fraction was immobile (Suppl. Note 2).

To assess the overlap, we selected a representative area of the cell, where no major bright or empty areas were present, and tracked the number and positions of the spots in the green and red channels using an automated tracking algorithm [23]. We restricted the analysis to the first two fully illuminated frames, because photobleaching reduced the co-localization in later frames. Spots where the position in the two channels differed by 213 nm or less were classified as overlapping and referred to as 'yellow' in the following. The cutoff distance of 213 nm was determined from *bona fide* yellow spots in the SNAP549-PDGFRTM-GFP construct (Suppl. Note 3). The average fraction of molecules in yellow spots was 12 ± 7% (s.d., *n* = 16 cells) for δOR, 13 ± 6% for κOR (*n* = 16), 13 ± 6% (*n* = 15) for µOR, and 12 ± 10% (*n* = 6) for the monomeric control. None of the OR yellow fractions is significantly different from the monomeric control (δOR: *p* < 0.99; κOR: *p* < 0.42; µOR: *p* < 0.52). We observed a linear dependence of the yellow spot fraction on the receptor density, which supports the view that they were indeed a result of coincidental vicinity of non-interacting green and red spots (Suppl. Fig. 2A). Importantly, the parameters of linear fits of the yellow-spot fraction to the spot density were not significantly different for the three ORs and the monomeric control and matched the analytical prediction of non-interacting proteins. Also, a Monte Carlo (MC) simulation of non-interacting spots yielded a similar fraction of yellow spots (12 ± 8%, *n* = 50).

To confirm that imaging of dually labeled proteins would indeed allow the detection of dimers or higher-order oligomers, we also used the constitutively dimerized metabotropic glutamate receptor mGluR1, the AMPA receptor subunit GluA1, which forms a constitutive tetramer, and the construct SNAP-PDGFRTM-GFP, which mimics a constitutive heterodimer. For the mGluR1, we found that the fraction of molecules in the yellow spots was 35 ± 11% (*n* = 6), for the GluA1 44 ± 11% (*n* = 8), and for the SNAP549-PDGFRTM-GFP 57 ± 15% (*n* = 6) (Fig. 2C). The increasing degree of co-localization of green and red labeled receptor subunits from the monomeric control to mGluR1 and GluA1 demonstrates that single-molecule imaging can reveal interactions in membrane proteins, and is also able to discriminate between different multimerization states.

We next evaluated the time that green and red spots remained co-localized in consecutive frames of the movies (excluding events with only a single frame of co-localization). For δOR, we obtained 98 ± 74 ms (s.d., *n* = 763 spots), for κOR 96 ± 85 ms (*n* = 584), for µOR 105 ± 94 ms (*n* = 581), for the monomer control 102 ± 63 ms (*n* = 178), and for the heterodimer mimic SNAP549-PDGFRTM-GFP 153 ± 158 ms (*n* = 1583). To further investigate possible mechanisms for the co-localization, we established a histogram of co-localization times. We used a semi-logarithmic presentation, where the rate constant of a decay process is visible as the histogram's slope (Fig. 2D). After normalization to match all histograms at their start, the histograms for the three ORs, the monomeric control and the MC simulation virtually coincided, and more importantly, had a constant slope over most of their range. This suggests that a single process is responsible for co-localization and the loss of co-localization in these experiments, which is the coincidental approach of green and red labeled receptors and their drifting apart due to diffusion (Fig. 2E). The time constant for loss of co-localization can be calculated from the estimate of the slope and is approximately 30 ms. In contrast to the ORs and the monomeric control, the time constant was about 120 ms for the heterodimer mimic SNAP549-PDGFRTM-GFP. This supports the view that here, a different process is responsible for loss of co-localization (presumably photobleaching or blinking of one of the fluorescent labels, or tracking errors due to crossing tracks).

Although the time for co-localization was low for the four protein pairs, we sometimes observed yellow spots for all three ORs (but not for the PDGFRTM monomeric control) where green and red co-localized much longer (Suppl. Fig. 3A and Suppl. Movie 3). However, since they occurred very rarely, they had no impact on the average time of co-localization.

In the single-molecule tracking and co-localization, we did not observe a larger fraction of yellow spots, or a longer time of co-localization for the κOR than for the δOR, µOR, or the monomeric control, in contrast to κOR’s higher GFP-positive fraction in the split-GFP assay. This suggests that under the low expression conditions used here, there was no significant level of dimerization for any of the proteins studied, and that the observed overlap was due to random co-localization without direct interaction.

### Single-molecule imaging of highly expressing cells with PhotoGate reveals κOR dimers

In the split-GFP assay at densities from 20–50/µm^2^, we observed a higher GFP-positive fraction for κOR than for δOR, µOR, and the monomer control; but in the single-molecule experiments, there was no significant amount of κOR dimers at densities < 5/µm^2^. Consequently, we should expect a transition from monomers to dimers in the range between these densities. To observe this transition and determine the dissociation constant, we require an assay that allows a quantification of the dimer fractions at higher densities, ideally up to 50/µm^2^ or above.

**Fig. 3:**
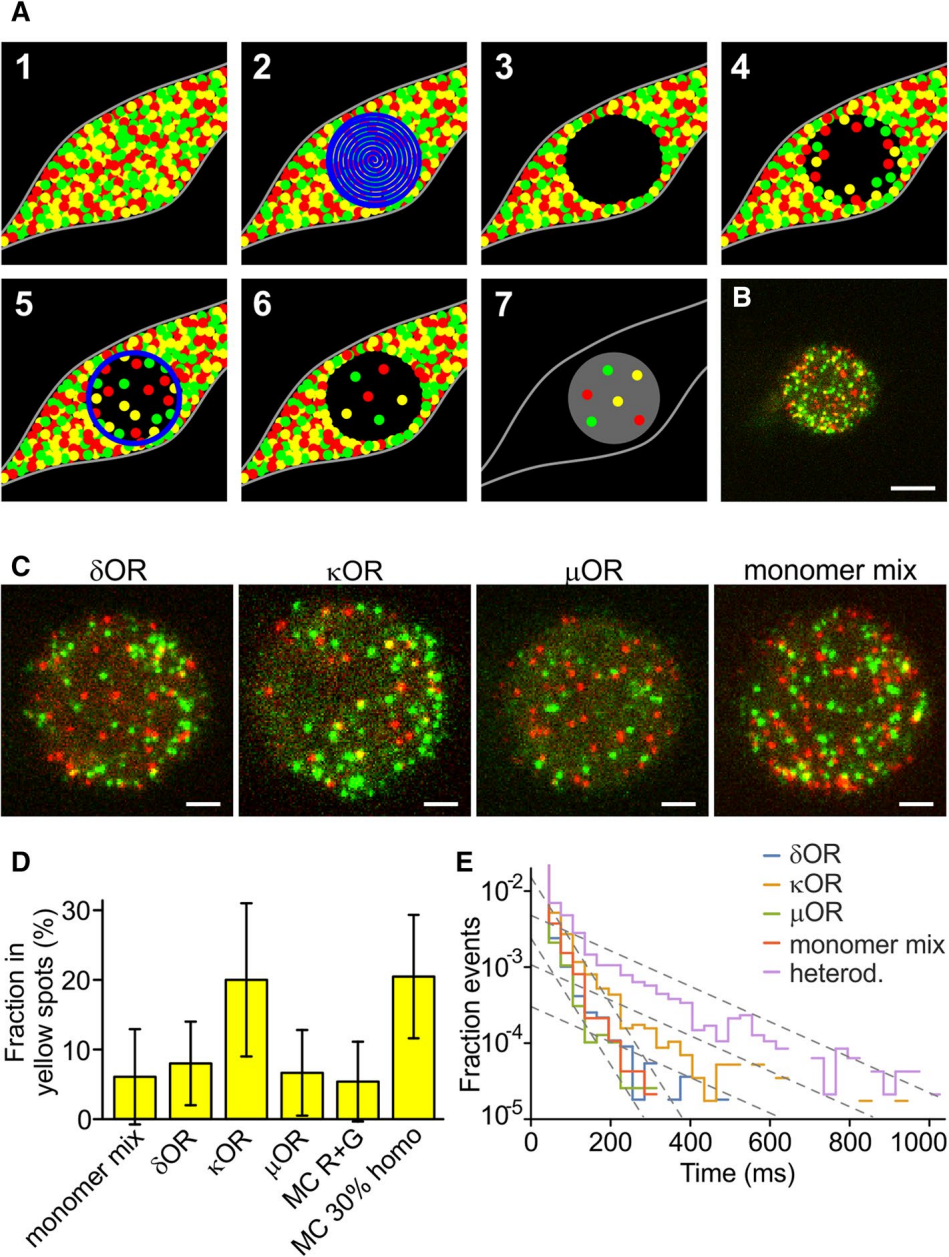
OR co-localization imaged with PhotoGate. **A** Schematic of the PhotoGate assay [15]. (1) A cell with a very high membrane density of fluorescent molecules (2) gets bleached in the central part, (3) leaving a dark bleached area. (4) After a while, the bleached area gets re-populated. (5) Bleaching a ring every few seconds prevents over-population by diffusion of unbleached molecules from the edges. (6) The result is a low density in the central area. (7) For imaging, only the central part gets illuminated. **B** First frame of a dual-color PhotoGate movie for κOR. Scale bar 5 µm. **C** Magnified central area for the ORs and the monomer mix (co-expressed SNAP549-PDGFRTM and PDGFRTM-GFP). For κOR, more yellow spots are visible. Scale bars 2 µm. **D** Fraction in yellow spots for the three ORs and the monomeric control. **E** Time of co-localization of the green and red fluorescence in a yellow spot

Two techniques called TOCCSL (Thinning Out Clusters while Conserving the Stoichiometry of Labeling) and PhotoGate were previously designed for this purpose [8, 18] (Fig. 3A). In a highly expressing cell, a part of the cell membrane gets photobleached quickly. After a recovery time, unbleached molecules re-populate the bleached area by lateral diffusion. The resulting density in the bleached area will be lower than the initial density. If the time allowed for recovery is shorter than the time for protein complexes to dissociate, the intact complexes can be imaged at lower density in the thinned-out region. In the PhotoGate technique, in addition to the initial bleaching exposure, a thin ring of high intensity is projected at certain time intervals to keep the density in the central imaging area low. To reduce glare from the high density, i.e., unbleached, part of the cell, an iris is used to restrict the illumination used for imaging to the low-density area.

We used the same constructs as for the previous experiment, i.e., we co-expressed X-GFP and SNAP549-X, where X was δOR, κOR, µOR, or PDGFRTM. However, this time we selected cells expressing a high density of molecules. In a small circular area of the cell with 10–15 µm diameter, GFP and SNAP549 were completely bleached with a focused laser beam. Then the laser was off for 5–20 s, depending on the expression level of the cell, to let molecules diffuse into the bleached area. Several rings were drawn at the outer edge of the bleached area to control the amount of recovery while allowing molecules inside the ring to distribute evenly. Finally, a two-color movie of the central area was recorded (Fig. 3B, C). Eventually, the remainder of the cell was imaged to determine the intensity per area and, by comparison to the unitary intensity of a single molecule, to calculate the density of molecules in the cell membrane.

As in the previous experiment, the overlap was assessed by automatically selecting green and red spots and classifying those spots as yellow where center positions were separated by the threshold distance of 213 nm or less. The average fraction of receptors in yellow spots was 8.0 ± 6.0% (s.d., *n* = 28 cells for δOR (avg. density in the unbleached cell 56 ± 37/µm^2^), 20.0 ± 11.0% for κOR (*n* = 18, 53 ± 35/µm^2^), 6.7 ± 6.1% (*n* = 23, 48 ± 35/µm^2^) for µOR and 6.1 ± 6.8% (*n* = 20, 59 ± 32/µm^2^) for the monomeric control (Fig. 3D and Suppl. Movies 4–7). At high membrane densities, the κOR shows a significantly higher co-localization of green and red than the monomeric control (*p* < 0.00006), while δOR (*p* < 0.3) and µOR (*p* < 0.7) do not. The dependence of the yellow spot fraction on the density in the imaged region was similar for δOR, µOR, the monomeric control and a MC simulation of non-interacting green and red spots (Fig. 3D and Suppl. Fig. 2B). However, the κOR showed a different behavior, which resembled a MC simulation of a homomeric protein where 35% of receptors resided in dimers and 65% in monomers.

To investigate the co-localization times of the ORs and the mechanisms involved, we analyzed the PhotoGate data in the same way as the initial single-molecule experiment without PhotoGate. We also imaged the construct SNAP549-PDGFRTM-GFP, where both tags are fused to the same protein (Fig. 3E). In the semi-exponential presentation, the histograms for δOR, µOR, and the monomer control (co-expressed SNAP549-PDGFRTM and PDGFRTM-GFP) again decay with a similar slope as for the experiment with low density, yielding a time constant for the loss of co-localization of 30 ms. The heterodimer mimic SNAP549-PDGFRTM-GFP also displays an initial decay with a similar slope, but then transitions into a slower decay of about 120 ms. For the κOR, we observe a similar behavior, with the slower decay starting a bit lower, but then continuing parallel to the decay of the heterodimer mimic. For SNAP549-PDGFRTM-GFP and κOR, we also observed multiple very long co-localizations (Suppl. Fig. 3B). The histograms for the heterodimer mimic and κOR can be interpreted as a superposition of two populations that have different mechanisms for the loss of co-localization. The faster process is the previously observed drifting apart due to diffusion. The other process, which is significantly slower, therefore, is most likely the photobleaching or blinking of one of the tags, which causes the co-localization to end. The similarity of the slopes for the heterodimer mimic and κOR suggests that the mechanism is the same for both proteins.

The large fraction of yellow spots that shows a fast, diffusion-based loss of co-localization can be explained by co-localizations of partially photobleached complexes that appear in later frames of the 100–200 frame long movies. When we restricted the analysis to spots that were present in the first illuminated frame, the initial part of the histogram with the steeper slope nearly disappeared for the heterodimer mimic SNAP549-PDGFRTM-GFP, and became much smaller for κOR, while δOR and µOR still only showed the steep decay (Suppl. Fig. 3C).

We again observed a small fraction of immobile spots (around 5%, Suppl. Note 2), and wanted to test whether they contributed a major part to the yellow spots and the events with a longer lifetime. However, the yellow spots were distributed between mobile and immobile fraction in proportion to the fraction size, meaning that there was no correlation between yellow and immobile spots. After excluding the immobile spots, neither the fraction of yellow spots nor the lifetimes changed notably (Suppl. Fig. 4).

These results support our assumption that an increased density shifts the equilibrium from monomers to dimers or higher-order clusters for κOR. Since monomeric control, δOR, and µOR have no tendency to dimerize or cluster, they stay monomeric. Also, since there was a delay of 5–20 s between photobleaching and imaging, κOR dimers or clusters must be stable for at least 5 s, because with faster dissociation, we would not have observed any interactions.

### Dissociation constant of κOR

**Fig. 4:**
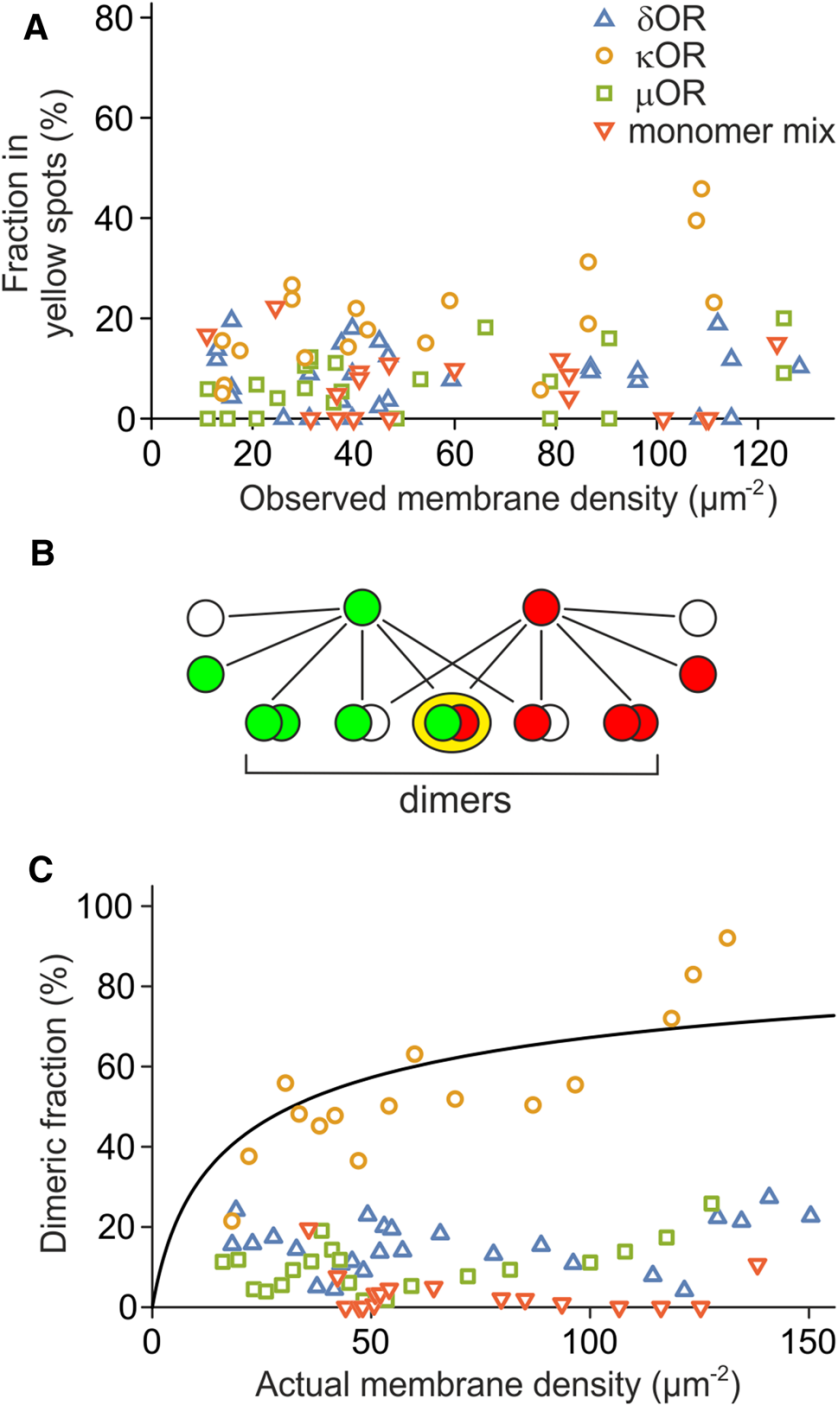
Dimerization of κOR. **A** The fraction of molecules in the yellow spots increases with density for κOR (green circles) but stays constant for δOR (red triangles), µOR (blue squares), and the monomer mix (grey crosses). **B** The dimeric fraction is much larger than the fraction of molecules in yellow spots due to the non-fluorescent fraction of GFP, the unlabeled SNAP-tag, and green/green and red/red dimers. **C** The dimeric fraction of κOR increases up to 100%, but the dimeric fractions for δOR and µOR stay in the low range of the monomeric control. Four consecutive data points were averaged to make the trend clearer. The fit of a binding curve (black line) to κOR yields the dissociation constant *k*_d_ = 32 ± 15/µm^2^

Finally, we set out to estimate the dissociation constant for κOR by analyzing how far the co-localization depended on the receptor density before reducing it by photobleaching. Therefore, we plotted all values of the yellow fraction obtained from the PhotoGate experiments as a function of total spot density before photobleaching (Fig. 4A). It is important to note that the density before photobleaching is not related to the spot density during imaging (which happens after photobleaching) and is up to 100 × higher. We observe that for δOR, µOR, and PDGFRTM, there is no trend for a higher yellow fraction at high densities. However, for κOR, the yellow fraction increases from a low value of 10% at a density of 14/µm^2^ to a high value of 46% for densities of > 100/µm^2^, supporting the expected density dependence of the κOR dimer/cluster fraction. As supported by previous studies on GPCRs and ORs, we assume in the following that the κOR is either monomeric or dimeric [2–7, 11–14].

The value shown in Fig. 4A is not the dimer fraction, but the fraction of yellow spots, which is not the same. While the fraction of yellow spots is limited due to green/green and red/red dimers and complexes containing non-fluorescent tags, we would expect the dimer fraction to increase further with density, reach values beyond 50%, and to asymptotically approach 100%. To calculate the dimer fraction from the fraction of yellow spots, we established a model that accounts for random co-localization, monomer and dimer fractions, non-fluorescent GFP and unlabeled SNAP-tag, and the green/green and red/red dimers (Fig. 4B and Suppl. Note 4). We measured the fraction of non-fluorescent GFP and the fraction of unlabeled SNAP-tag in an experiment with SNAP549-PDGFRTM-GFP. Since every molecule carries both tags, the non-fluorescent/unlabeled fractions can directly be determined from the fraction of green, red, and yellow spots (Suppl. Note 5). The fraction of fluorescent GFP was 54 ± 3%, the fraction of labeled SNAP-tag was 62 ± 2% (*n* = 15, s.e.m.).

The model allowed us to calculate the fraction of receptor subunits in dimers from the numbers of yellow, green, and red spots, and the density during imaging (Suppl. Fig. 5). We find that for the κOR, the dimer fractions increase up to 69%, while for δOR and µOR, they remain on a lower level similar to the monomeric control. This behavior becomes more visible when reducing variability by averaging several data points (Fig. 4C). We then set out to find a dissociation constant that allows a common best fit of the model parameters to the experimentally obtained data. The dissociation constant is an implicit parameter in the model, and is related to the fraction of receptors in dimers *f*_d_ by the following equation:

$$f_{{\text{d}}} = 1 + \left( {k_{{\text{d}}} - \sqrt {k_{{\text{d}}} \left( {k_{{\text{d}}} + 8\left[ R \right]} \right)} } \right)/4\left[ R \right],$$

with the dissociation constant *k*_d_ and the receptor density [*R*], which we corrected for non-fluorescent GFP and unlabeled SNAP-tag (Suppl. Note 6). For the κOR, a nonlinear fit yielded the dissociation constant *k*_d_ = 32 ± 15/µm^2^ (68% confidence interval) (Suppl. Note 7). Similar attempts to determine dissociation constants for the other proteins yielded (2.1 ± 2.7)·10^3^/µm^2^ for δOR, (1.9 ± 4.3)·10^3^/µm^2^ for µOR and $\infty$ for the monomeric control (Suppl. Fig. 5). Taken together, we conclude that κOR forms dimers at expression levels above 20/µm^2^, but δOR and µOR stay mainly monomeric, at least at densities up to 100/µm^2^.

## Discussion

In this study, we used a split-GFP complementation assay, conventional single-molecule imaging, and the PhotoGate single-molecule technique to assess the dimerization of ORs. We find that κOR forms homodimers at densities below 100/µm^2^, while δOR and µOR stay mainly monomeric. The dissociation constant for κOR is *k*_d_ = 32 ± 15/µm^2^. We observe that co-localization of green and red labeled receptors of the δOR, the µOR and the monomeric control is only transient, whereas κOR shows longer lasting interactions at the same densities. We also considered the possibility that co-localization was caused by clustering at cellular structures, e.g., clathrin coated pits; in this case, we would expect immobilization of the spots. However, the immobile fraction of spots was smaller for the experiment with PhotoGate than for the experiment without PhotoGate, suggesting that the co-localization was not caused by clustering at cytoskeletal structures or internalization sites (Suppl. Note 2).

In general, it is difficult to measure lateral affinities of membrane proteins because of the inability of biochemical methods to determine the density of the target protein in the plasma membrane. So far, this can only be achieved with fluorescence-based assays where the signal from the labeled protein in the membrane of a living cell can be compared to a reference of known concentration or a direct observation of single molecules is possible. With conventional single-molecule imaging, maximum densities of 5/µm^2^ are accessible [9, 10]. With bulk fluorescence methods using confocal microscopy, the minimum measured densities were 160/µm^2^ [24–26]. The PhotoGate approach we used covers the missing range of 10–100/µm^2^. It remains to be seen if measurements from the three ranges can be consolidated into a common framework.

A major contribution to the error in the dissociation constant originates—as for many single-molecule methods—from the uncertainty inherent to counting low numbers of events, in our case the green, red, and yellow spots. The uncertainty in the initial counts (Fig. 4A) gets even amplified when subtracting the expected number of yellow spots caused by random co-localization (Fig. 4C). A second error source that is difficult to account for is the variability of the SNAP-tag labeling efficiency. In the model to calculate the fraction of dimers from the fraction of yellow spots, we used the average SNAP-tag labeling efficiency, but the actual labeling efficiency might vary from one cell to another, e.g., due to different accessibility of the substrate in the extracellular solution to the cell (see also Suppl. Note 5).

A possible source for a systematic error that is more difficult to compensate lies in the design of the PhotoGate technique. A delay is required between the initial bleaching and the movie acquisition to allow molecules from the outside to diffuse into the bleached center (Fig. 3A). In our experiments, this delay was in the range of 5–20 s. If receptor dimers dissociate in a comparable time frame, then a significant fraction of the yellow dimers will have decayed by the time of imaging and will, therefore, appear as only green or only red. If they dimerize again, the chance to find a fluorescent partner is low, because most proteins in the imaging area are bleached. However, we estimate that this effect reduces the fraction of yellow dimers by less than 10% in the case of the κOR since we obtained a large yellow dimer fraction for membrane densities above 30/µm^2^. On the other hand, the fact that even after a long delay of up to 20 s κOR dimers remain intact, means that the κOR dissociation time is larger or at least in the range of 20 s. In principle, it is possible that the yellow δOR and/or µOR dimers are depleted by fast dissociation (at least faster than 5 s), and that our interpretation of δOR and µOR to be monomers at all tested densities is incorrect. However, one would then expect the GFP-positive fraction in the split-GFP assay to be larger than what we observed. Therefore, we think that our experiments are consistent with δOR and µOR being primarily monomers at the densities we observed.

For many other in vitro studies, the receptors were expressed under control of the strong CMV promoter; in contrast, we here used a promoter with inducible expression and selected cells with receptor densities of 200/µm^2^ or less. To investigate whether much higher expression levels could be a possible cause for finding dimers for all ORs (not only κOR) with biochemical or other bulk methods, we measured the membrane densities of δOR when expressed under the CMV promoter. We found that although the majority of cells expressed less than 500 receptors/µm^2^, receptor densities reached up to 6000/µm^2^ (Suppl. Fig. 6). But since the higher expressing cells contain more receptors, only a small fraction of 4% of the receptors experience densities below 500 receptors/µm^2^, and more than half of the receptors reside at densities above 3000/µm^2^. Accordingly, bulk experiments (e.g., BRET and co-IP) will mainly yield the signals from the small fraction of high-density cells. Our observation that δOR and µOR are monomeric at low densities is, therefore, consistent with other studies if the dissociation constants of δOR and µOR lie above 200/µm^2^.

Some current sources offer data on RNA levels in all organs, which suggest that ORs are expressed in most tissues, but at strongly differing levels [27]. However, it was not determined whether just a small fraction of cells express the receptors strongly, or most of the cells at low or moderate levels. Accordingly, expression levels and membrane densities in individual cells remain unknown. To understand the impact of OR dimerization in vivo, receptor membrane densities in different cell types in the body need to be measured in the future.

## Materials and methods

### DNA constructs

The murine δ- and κ-opioid receptor, rat µ-opioid receptor, and the transmembrane domain of PDGFRα were cloned into the pWHE636 vector (gift by Christian Berens) that contains a tetracycline inducible promoter [28]. An N-terminal signal peptide and the SNAP-tag were fused to the N termini and GFP to the C-termini of the receptors and PDGFRTM with flexible linkers. To have well-folding, soluble domains on both sides of PDGFRTM, we added a non-fluorescent GFP carrying the mutation Y66l (to render it non-fluorescent) to the C-terminus of SNAP-PDGFRTM, and a signal peptide, followed by a HALO-tag, to the N-terminus of PDGFRTM-GFP. For split GFP, β-sheets 1–10 (aa 1–214) of a GFP variant were fused to the constructs with the SNAP-tag, and β-sheet 11 (aa 215–230) and mKate to the constructs without SNAP-tag [21].

### Expression of ORs in CHO cells

For inducible expression in mammalian cells, a stable CHO-K1 cell line (ATCC) was made with the regulator plasmid pWHE644 containing a tetracycline-regulated transactivator and a transcriptional silencer [28]. The cells were seeded on a high refractive index coverslip (*n* = 1.78) and grown in complete DMEM for 12–15 h to obtain 60–80% confluency before transfection with polyethylenimine (1 mg/mL) with 1 µg of each DNA per coverslip. After 3 h, cells were washed in DPBS and incubated with doxycycline in DMEM to induce expression. The induction was terminated by washing with DPBS, and then complete DMEM was added to cells. Imaging started after 12–15 h of induction of expression. For single-molecule imaging and split-GFP assay, cells were incubated for 2 h with 1 µg/mL of doxycycline hyclate, for the PhotoGate experiment for 3 h with 2 µg/mL. For SNAP-tag labeling, cells were incubated with 2 nM benzylguanine-Alexa Fluor 647 (New England Biolabs, SNAP-Surface Alexa Fluor 647) or custom synthesized SNAP substrate benzylguanine-DY-549P1 for 30 min before an experiment, and unbound dye was washed away by rinsing 5 × with DPBS.

### Dye synthesis

HPLC analysis (1 mL/min) and purification (3 mL/min) were performed on an Agilent Technologies 1260 Infinity system using UV detection at 290 nm and—for analysis—a Phenomenex Kinetex^®^ 5u XB-C18 100 Å 250 × 4.6 mm column or—for purification—a Phenomenex Synergi^®^ 10u Hydro–RP 80 Å 250 × 15.0 mm column. Eluent A was water containing 0.05% trifluoroacetic acid (TFA) and eluent B was acetonitrile containing 0.05% TFA. Linear gradient conditions were as follows: 0–1 min, A/B (90:10); 1 − 21 min, linear increase to 100% of B; 21 − 23 min, 100% B; 23–23.3 min: A/B (90:10); 23.3–26 min: A/B (90:10). Characterization was performed through mass spectrometry and mass spectra were recorded on a Thermo Scientific Exactive mass spectrometer using electrospray ionization (ESI) as ion sources.

To a stirred solution of 6-((4-(aminomethyl)benzyl)oxy)-7*H*-purin-2-amine (1.4 mg, 5.2 µmol, 1.1 eq.) in CH_3_CN (250 µL), under inert atmosphere, was added the NHS ester of DY-549P1 (5 mg, 4.8 µmol, 1 eq.) in 250 µL of H_2_O, followed by NaHCO_3_ (50 µL of a 1 m solution: final concentration of c.a. 0.1 M) and the reaction was stirred protected from light for 48 h. After completion (RP-C18 TLC:H_2_O/CH_3_CN 3:7) the solvent was removed to give a pink oil. The crude SNAP549 was then dissolved in the minimum volume of methanol, and purified using preparative HPLC (see conditions above). The product was isolated as a pink oil (3.3 mg, 55% yield). C_49_H_57_N_8_Na_3_O_15_S_4_ (1195.25 g/mol). HPLC: t_R_ = 6.935 min (82% purity—18% of DY-549P1-OH remaining). ESI-HRMS (–): m/z calcd for C_49_H_56_N_8_Na_3_O_15_S_4_: 1193.2447 [M–H]^–^; found 1193.2434 [M–H]^−^; m/z calcd for C_49_H_57_N_8_Na_2_O_15_S_4_: 1171.2627 [M–Na]^−^; found 1171.2611 [M–Na]^−^; m/z calcd for C_44_H_54_N_3_Na_2_O_15_S_4_: 1038.2239 [M–2AP–Na]^−^; found 1038.2229 [M–2AP–Na]^−^.

### Microscopy

Imaging was done on an Olympus IX71 base equipped with an Olympus 100 × NA1.70 objective, a back-illuminated EMCCD camera (Andor iXon DV-897 BV), an emission filter wheel and an OptoSplit II beam splitter (Cairn). GFP, mKate/BG549, and BG647 were excited through a custom built total internal reflection illumination pathway either consecutively (split-GFP assay) or in alternating excitation (single-molecule movies). Movies (256 × 256 pixels) were recorded at a frame rate of 17 or 33 Hz or, for alternating excitation, at a frame rate of 65 Hz. Single-molecule imaging was done at power densities of 100–250 W/cm^2^. During the experiments, cells with similar expression levels in all channels were chosen. For single-molecule imaging without PhotoGate, cells with expression < 5/µm^2^ were selected.

### PhotoGate

The PhotoGate assay was done similarly as described in [18]. A 473 nm laser beam (25 mW before the objective) focused on the plane of the plasma membrane was directed using a galvo scanning system placed at an appropriate position in the light path. Despite 473 nm being far from the excitation maximum for DY-549P1, the intensity of the laser was sufficiently high to photobleach the dye completely. A region of interest of diameter 10–15 µm was bleached for about 10 s, depositing a total energy of 2.5 mJ/µm^2^, by moving the focused beam in a spiral motion. After a 5–20 s delay (depending on fluorophore density), 2–8 rings were drawn on the cell's surface at intervals of 3–5 s, to control the density inside the pre-bleached area. Right after, an iris in the illumination pathway was closed to restrict the illumination to the central area and eliminate glare from the bright area outside, and the movie acquisition was started.

### Analysis of single-molecule images

The first two fully illuminated frames for each color were analyzed. A rectangular region of interest was chosen that was completely covered by plasma membrane and had an even distribution of spots. The center positions of green and red spots were automatically selected by the MOSAIC tracking suite [23]. The fraction of receptors in yellow spots was calculated as $f_{{\text{d}}} = 2N_{{\text{y}}} /(2N_{{\text{y}}} + N_{{\text{g}}} + N_{{\text{r}}} )$. When the centers of green and red spots were as close as 213 nm or less, both were combined to form a yellow spot. For determination of co-localization time constants, first green and red trajectories were identified by the MOSAIC tracking plugin with an allowed frame-to-frame movement of a spot of 500 nm. Green and red trajectories were overlaid, and a yellow trajectory was started if a green and red trajectory came closer than 213 nm. The yellow trajectory was stopped as soon as the distance became larger than 213 nm or one of the trajectories ended. Co-localization time constants were estimated by adjusting the slope of a line in the semi-logarithmic histograms of yellow trajectory lengths. Averaging of data points for Fig. 4C accounts for the number of spots in each individual experiment using the sum of red, green and yellow spots as a weight in the average. All significance levels were calculated using the Mann–Whitney *U* test for pairs of data sets, e.g., the PDGFRTM monomeric control and one of the ORs.

### Density evaluation

For calculating the density of fluorescent receptors in the split-GFP and the PhotoGate assay, the intensities of single molecules were compared to the intensities of a region of interest. First, a small region of interest (0.5 × 0.5 µm^2^) around individual fluorescent molecules (either from a lower-expressing cell or after photobleaching of the majority of spots) was selected, and background from a nearby region without a spot or the same region after photobleaching of the spot was subtracted. An average value was formed from 10–20 spots. Similarly, for an area with high density, background was selected from an area outside the cell.

## Supplementary Information

Below is the link to the electronic supplementary material.

Supplementary file1 (MP4 1401 KB)

Supplementary file2 (MP4 355 KB)

Supplementary file3 (MP4 393 KB)

Supplementary file4 (MP4 1153 KB)

Supplementary file5 (MP4 821 KB)

Supplementary file6 (MP4 1100 KB)

Supplementary file7 (MP4 1151 KB)

Supplementary file8 (DOCX 2110 KB)

## Data Availability

Material (DNA, cells, dye) and raw data generated during the current study are available from the corresponding author on reasonable request.

## Code availability

Data were evaluated using Go (open source), LabView (National Instruments), Mathematica (Wolfram Research), and Excel (Microsoft) using custom code. Code can be made available by the corresponding author on reasonable request.
